# Structural Insights for Activation of Retinal Guanylate Cyclase by GCAP1

**DOI:** 10.1371/journal.pone.0081822

**Published:** 2013-11-13

**Authors:** Sunghyuk Lim, Igor V. Peshenko, Alexander M. Dizhoor, James B. Ames

**Affiliations:** 1 Department of Chemistry, University of California Davis, Davis, California, United States of America; 2 Basic Sciences, Pennsylvania College of Optometry, Salus University, Elkins Park, Pennsylvania, United States of America; University of Oldenburg, Germany

## Abstract

Guanylyl cyclase activating protein 1 (GCAP1), a member of the neuronal calcium sensor (NCS) subclass of the calmodulin superfamily, confers Ca^2+^-sensitive activation of retinal guanylyl cyclase 1 (RetGC1) upon light activation of photoreceptor cells. Here we present NMR assignments and functional analysis to probe Ca^2+^-dependent structural changes in GCAP1 that control activation of RetGC. NMR assignments were obtained for both the Ca^2+^-saturated inhibitory state of GCAP1 versus a GCAP1 mutant (D144N/D148G, called EF4mut), which lacks Ca^2+^ binding in EF-hand 4 and models the Ca^2+^-free/Mg^2+^-bound activator state of GCAP1. NMR chemical shifts of backbone resonances for Ca^2+^-saturated wild type GCAP1 are overall similar to those of EF4mut, suggesting a similar main chain structure for assigned residues in both the Ca^2+^-free activator and Ca^2+^-bound inhibitor states. This contrasts with large Ca^2+^-induced chemical shift differences and hence dramatic structural changes seen for other NCS proteins including recoverin and NCS-1. The largest chemical shift differences between GCAP1 and EF4mut are seen for residues in EF4 (S141, K142, V145, N146, G147, G149, E150, L153, E154, M157, E158, Q161, L166), but mutagenesis of EF4 residues (F140A, K142D, L153R, L166R) had little effect on RetGC1 activation. A few GCAP1 residues in EF-hand 1 (K23, T27, G32) also show large chemical shift differences, and two of the mutations (K23D and G32N) each decrease the activation of RetGC, consistent with a functional conformational change in EF1. GCAP1 residues at the domain interface (V77, A78, L82) have NMR resonances that are exchange broadened, suggesting these residues may be conformationally dynamic, consistent with previous studies showing these residues are in a region essential for activating RetGC1.

## Introduction

Guanylyl cyclase activating proteins (GCAPs) belong to the neuronal calcium sensor (NCS) branch of the calmodulin superfamily [1–3] and regulate Ca^2+^-sensitive activity of retinal guanylyl cyclase (RetGC) in rod and cone cells [4–6]. Phototransduction in retinal rods and cones is modulated by intracellular Ca^2+^ sensed by GCAPs [7,8] and defects in Ca^2+^ signaling by GCAPs are linked to retinal diseases [9]. Light excitation of photoreceptor cells triggers a phototransduction cascade that causes hydrolysis of cGMP and hence closure of cGMP-gated channels [10]. Light-activated channel closure blocks the entry of Ca^2+^, which lowers the cytosolic Ca^2+^ concentration from ~250-500 nM in the dark down to ~25 nM in the light [11]. This drop in Ca^2+^ causes the change in formation of Ca^2+^-free/Mg^2+^-bound GCAPs that activate RetGC [12], whereas Ca^2+^-bound GCAPs inhibit RetGC at high Ca^2+^ levels maintained in the dark [13–15].

**Figure 1:**
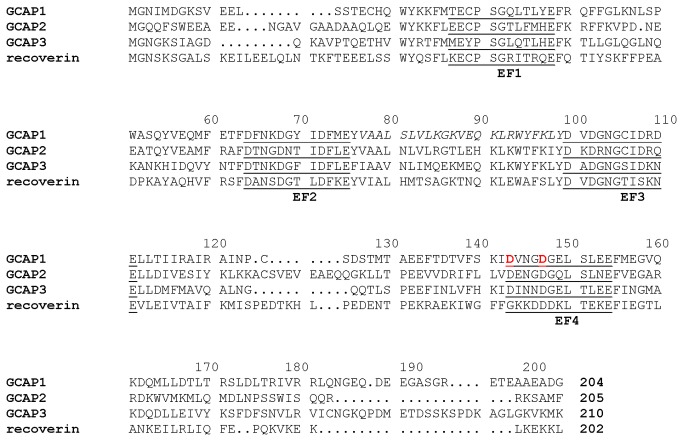
Amino acid sequence alignment of GCAP1 with various NCS proteins. Secondary structural elements are indicated schematically. The four EF-hands (EF1, EF2, EF3 and EF4) are underlined. Residues mutated in EF4mut (D144N/D148G) are indicated in red. Residues at the domain interface (V77 – L97) that have broadened NMR resonances are shown in italics.

The GCAPs (GCAP1 [6], GCAP2 [16], GCAP3 [17] and GCAP4-8 [18]) are all ~200-amino acid residue proteins containing a covalently attached N-terminal myristoyl group and four EF-hand motifs (EF1 through EF4, Figure 1). Mg^2+^ binds to three EF-hands (EF2, EF3 and EF4) when cytosolic Ca^2+^ levels are low and Mg^2+^-bound GCAP1 activates RetGC, preferentially its RetGC1 isozyme [12,19,20]. The X-ray crystal structure of Ca^2+^-bound GCAP1 [21] and NMR structure of GCAP2 [22] showed that the four EF-hands form two semi-globular domains (EF1 and EF2 in the N-domain, and EF3 and EF4 in the C-domain); Ca^2+^ is bound at EF2, EF3 and EF4; and the N-terminal myristoyl group in GCAP1 is buried inside the Ca^2+^-bound protein, flanked by hydrophobic residues at the N- and C-termini (see italicized residues in Figure 1). The structure of the physiological activator form of GCAPs (Mg^2+^-bound/Ca^2+^-free state) is currently unknown. 

Recoverin is the only NCS protein whose structure is known in both the Ca^2+^-free and Ca^2+^-bound states (Figure 1) [23,24]. Ca^2+^-free recoverin contains a myristoyl group sequestered inside the protein that interacts intimately with residues from EF1, EF2 and EF3 [25,26]. Ca^2+^ binding at EF2 and EF3 leads to a 45°-swiveling of the two domains in recoverin that promotes extrusion of the fatty acyl group outward (termed Ca^2+^-myristoyl switch), enabling it to interact with membrane targets [23,27]. Previously, we have shown that GCAP1 does not possess a Ca^2+^-myristoyl switch because the attached myristoyl group in GCAP1 remains sequestered in both the Ca^2+^-free/Mg^2+^-bound and Ca^2+^-bound states [28]. However, we wondered if GCAP1 might undergo a Ca^2+^-induced rearrangement at the domain interface like what is seen for both recoverin [23] and NCS-1 [29]. Here, we present NMR and mutagenesis functional analysis on GCAP1 to probe structural changes between the Ca^2+^-saturated inhibitory state versus a GCAP1 mutant (D144N/D148G, called EF4mut), which contains Ca^2+^ bound at EF2 and EF3, but lacks Ca^2+^ at EF4 and was shown to serve as a functional mimic of GCAP1 in the Ca^2+^-free/Mg^2+^-bound activator state [12]. Our results indicate that EF4mut (activator) and Ca^2+^-saturated GCAP1 (inhibitor) have fairly similar backbone NMR chemical shifts. The largest chemical shift differences are seen for residues in EF4 and a few residues in EF1 (K23, T27 and G32). GCAP1 residues at the interface between EF2 and EF3 (V77, A78, L82 and W94) have broadened NMR resonances, indicating these residues are conformationally flexible in both activator and inhibitor states.

## Results

### NMR assignments for GCAP1 activator mutant (EF4mut)

**Figure 2:**
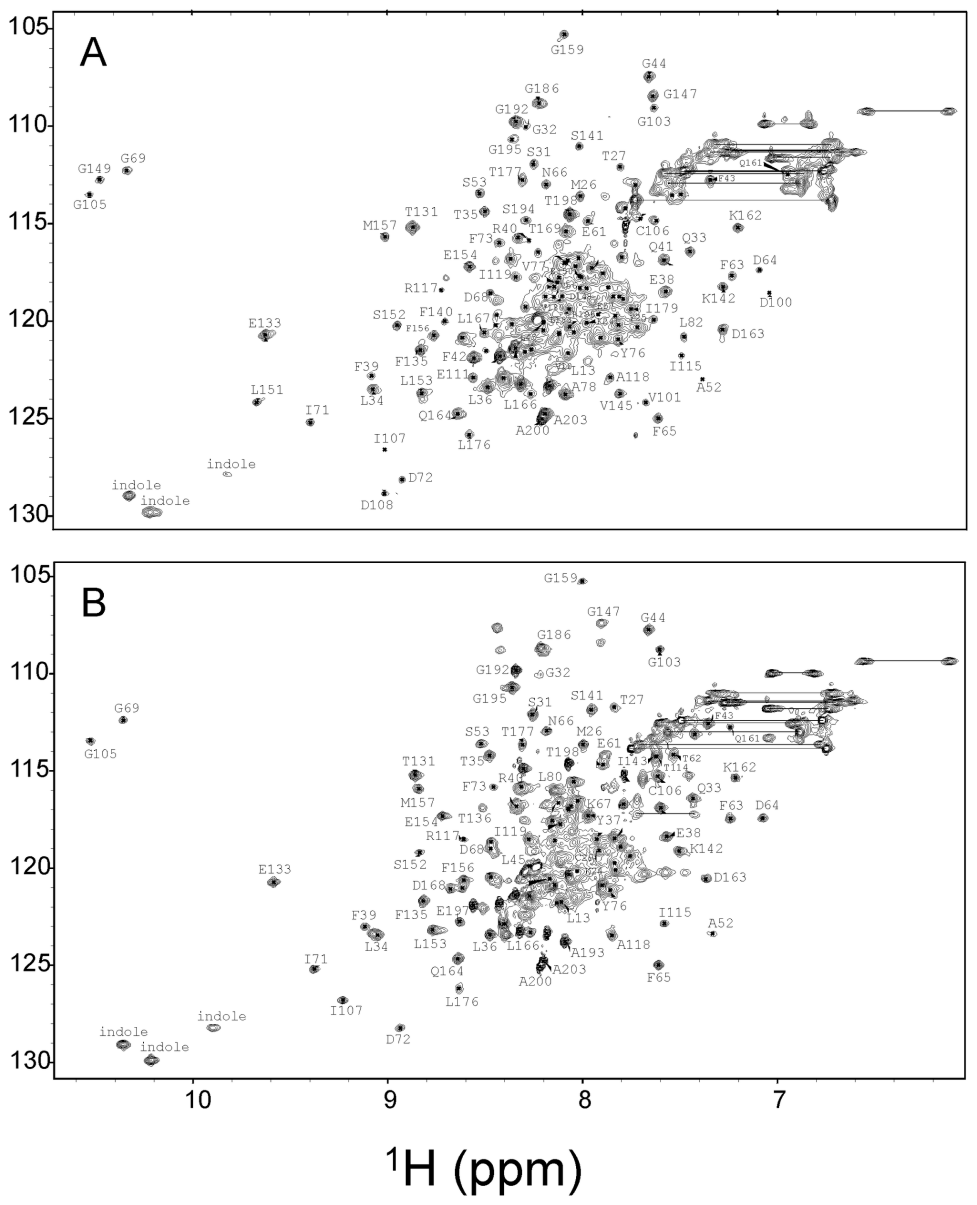
NMR spectra of activator vs. inhibitor forms of GCAP1. Two-dimensional (^1^H-^15^N HSQC) NMR spectra of ^15^N-labeled wildtype Ca^2+^-saturated GCAP1 (A) and EF4mut (B). Spectra were obtained at 37 °C in the presence of 40 mM octylglucoside. Downfield resonances (~10.5 ppm) are assigned to conserved glycine residues in each Ca^2+^-bound EF-hand loop. For Ca^2+^-saturated GCAP1 (A), downfield peaks assigned to G69, G105 and G149 indicate Ca^2+^ is bound at EF2, EF3 and EF4. For EF4mut (B), downfield peaks assigned to G69 and G105 indicate Ca^2+^ is bound at EF2 and EF3 in EF4mut. Sequence specific resonance assignments for Ca^2+^-saturated GCAP1 were determined previously [31].

NMR spectroscopy was used to probe Ca^2+^-induced protein conformational changes in GCAP1 by examining ^15^N-^1^H HSQC spectra of Ca^2+^-saturated wildtype protein (Figure 2A) and comparing it to that of the EF4mut activator state (Figure 2B). The EF4mut sample with Ca^2+^ bound at EF2 and EF3 (and no Ca^2+^ bound at EF4) was shown previously to activate RetGC and therefore serves as a functional mimic of the Ca^2+^-free/Mg^2+^-bound activator state [12]. The EF4mut sample was used rather than the wildtype Ca^2+^-free/Mg^2+^-bound activator state, because EF4mut is more soluble and stable under conditions for NMR (the Ca^2+^-free/Mg^2+^-bound wildtype protein tends to aggregate under NMR conditions, whereas EF4mut does not). Ca^2+^ binding to EF2 and EF3 in EF4mut activator state increases the folding stability that allows the protein to remain folded in NMR experiments performed at elevated temperatures (37 °C), which was necessary to achieve improved spectral quality. Although the NMR spectrum of EF4mut (with Ca^2+^ bound at EF2 and EF3) is much improved compared to that of Ca^2+^-free/Mg^2+^-bound wildtype, EF4mut still has somewhat broadened NMR peaks compared to what is expected for a monomeric protein at 23 kDa, suggesting that EF4mut might form a dimer under NMR conditions. 

**Figure 3:**
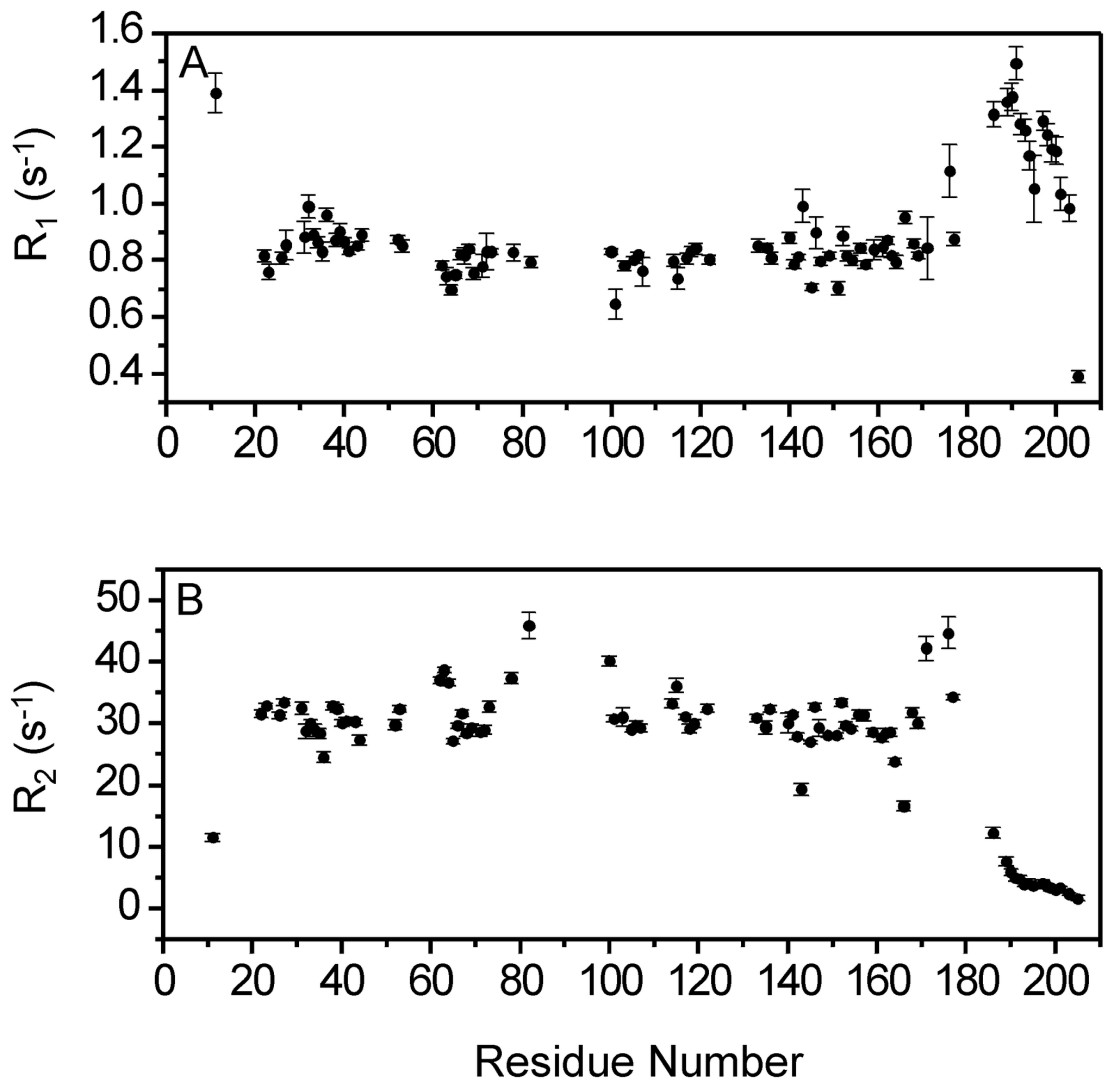
GCAP1 forms a dimer in solution. ^15^N NMR relaxation data are shown for EF4mut. Spin-lattice relaxation rate constants (A) and spin-spin relaxation rate constants (B) are plotted as a function of residue number. All data were measured at 60.81 MHz ^15^N frequency and 37 °C. (C) Size-exclusion chromatography (SEC) elution profiles are shown for GCAP1 wildtype (solid line) and V77E (dotted line). The protein concentration was 200 μM for the samples analyzed by SEC.

To verify whether GCAP1 forms a dimer in our NMR experiments, the rotational correlation time (τ_c_) of the protein was measured by ^15^N NMR relaxation analysis (Figure 3). The longitudinal and transverse ^15^N NMR relaxation rates (R_1_ and R_2_) for each assigned amide resonance of EF4mut are shown in Figure 3. The average ^15^N R_1_ and R_2_ values from residues in structured regions are 0.84 (± 0.04) s^-1^ and 28 (± 0.5) s^-1^, respectively. Assuming isotropic tumbling, the average rotational correlation time obtained from R_1_/R_2_ ratios of all residues within 1 standard deviation of the average value [30] was calculated to be 22 ±1 ns at 37 °C, consistent with an average molar mass of 36 ±5 kDa, which is considerably higher than the calculated mass of a protein monomer (23 kDa). In addition, size-exclusion chromatography analysis performed at high concentration of GCAP1 (Figure 3C) indicated that Ca^2+^-saturated wildtype GCAP1 has elution volume consistent with a molar mass of 40 ±4 kDa. EF4mut has the same elution volume as wildtype (not shown). Thus, both size-exclusion chromatography and NMR relaxation data suggest that Ca^2+^-saturated GCAP1 and EF4mut are at least partially dimeric under NMR conditions. 

The peaks in the ^1^H-^15^N HSQC NMR spectrum of GCAP1 (Figure 2) represent main chain and side-chain amide groups that provide a residue-specific fingerprint of the overall protein conformation. The resonance assignments for Ca^2+^-saturated GCAP1 were reported previously [31]. The NMR assignments for EF4mut were determined in this study as described in Methods. Because EF4mut forms a dimer under NMR conditions (Figure 3), the peak broadening due to dimerization caused a significant decrease in the signal-to-noise ratio and prevented detection of at least 25% of expected backbone resonances in three-dimensional NMR experiments. Therefore, backbone assignments were obtained for ~70% of the residues in EF4mut compared with ~85% assignment for Ca^2+^-saturated GCAP1 [31]. The unassigned residues for EF4mut (due to peak broadening) include the first 10 residues from the N-terminus, part of the exiting helix of EF2 (residues M74 – A79), domain linker (residues S81 – Q90), entering helix of EF3 (residues K91 – Y99), linker between EF3 and EF4 (residues N123 – M130), and part of the C-terminal helix adjacent to EF4 (residues L170 – D175). The broadening of these unassigned resonances in EF4mut suggests these regions in the activator state are conformationally dynamic. 

Downfield shifted NMR peaks at ~10.5 ppm for GCAP1 (inset, Figure 2) are assigned to conserved glycine residues in the EF-hand Ca^2+^-binding loops and are characteristic of Ca^2+^ bound EF-hands. In Ca^2+^-saturated GCAP1 (Figure 2A), three downfield peaks assigned to G69 (EF2), G105 (EF3) and G149 (EF4) confirm that Ca^2+^ is bound at EF2, EF3 and EF4 as seen in the crystal structure [21]. In EF4mut (Figure 2B), two downfield peaks assigned to G69 (EF2) and G105 (EF3) confirm that Ca^2+^ is bound at EF2 and EF3 (and not EF4) as seen by fluorescence spectroscopy of the mutants [12,19]. Thus, the binding of Ca^2+^ at EF4 in GCAP1 is critical for switching between the activator and inhibitor conformational states.

### Mapping chemical shift changes in activator vs inhibitor forms of GCAP1

**Figure 4:**
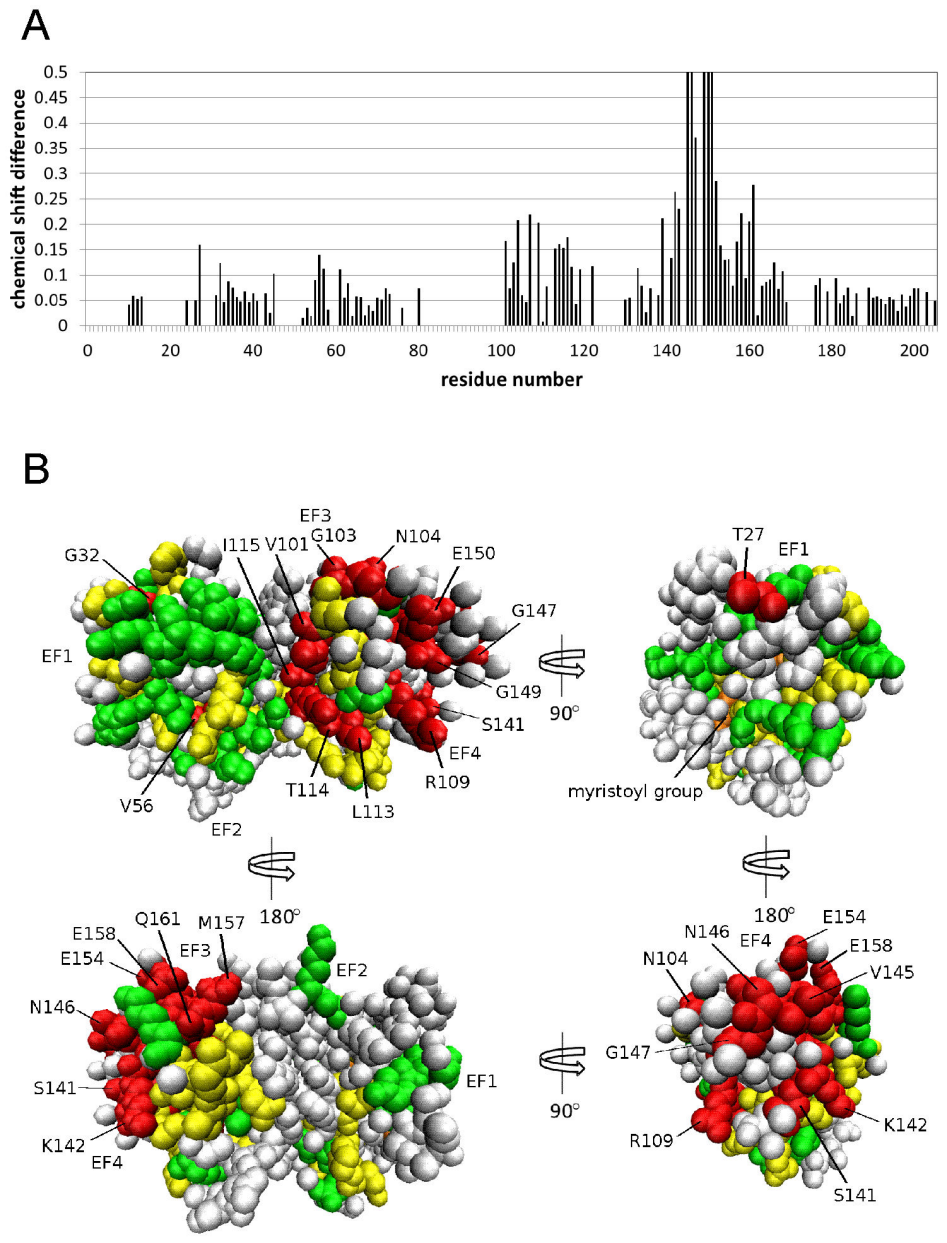
NMR chemical shift mapping for GCAP1. (A) Amide chemical shift differences between EF4mut and Ca^2+^-saturated wildtype (CSD = {(H_N_^A^ – H_N_^I^)^2^ + (^15^N^A^ – ^15^N^I^)^2^}^1/2^ , where “A” and “I” designate activator and inhibitor states) plotted as a function of residue number. (B) CSD values from part “A” are mapped onto the GCAP1 crystal structure (2R2I). Residues with the largest chemical shift difference (CSD > 0.12 ppm) are highlighted in red, intermediate chemical shift differences (0.06 < CSD < 0.12) shown in yellow, and smallest chemical shift differences (CSD < 0.06) shown in green. Unassigned residues are colored gray and the myristoyl group is orange.

The NMR chemical shift assignments in Figure 2 were used to calculate the chemical shift difference (CSD = {(H_N_^A^ – H_N_^I^)^2^ + (^15^N^A^ –^15^N^I^)^2^}^1/2^) for activator vs inhibitor forms of GCAP1 plotted as a function of residue in Figure 4A (where H_N_^A^ and ^15^N^A^ are amide ^1^H and ^15^N chemical shifts in the activator state, and H_N_^I^ and ^15^N^I^ are amide ^1^H and ^15^N chemical shifts in the inhibitor state). The average CSD value calculated from all assigned residues in GCAP1 (CSD_ave_ = 0.14 ppm) is 10-fold lower than that calculated for the two conformational states of recoverin’s Ca^2+^-myristoyl switch [23]. The relatively low CSD_ave_ is consistent with GCAP1 having a fairly similar main chain topology (for the assigned residues) in both EF4mut activator and Ca^2+^-saturated inhibitor states. The small structural change for the two states of GCAP1 is in stark contrast to the profound structural differences between the two conformational states of recoverin’s Ca^2+^-myristoyl switch [32,33]. The myristoyl group of recoverin is buried inside the protein in the Ca^2+^-free state, and Ca^2+^-binding to recoverin promotes extrusion of the myristoyl group with a concomitant change in protein structure [23]. By contrast for GCAP1, the attached myristoyl group is sequestered inside the protein in Ca^2+^-free, Ca^2+^-free/Mg^2+^-bound and Ca^2+^-saturated states [28], consistent with our finding that the overall main chain structure of GCAP1 remains similar for both the EF4mut activator and Ca^2+^-saturated inhibitor forms.

The GCAP1 residues having largest CSD values are found in EF4 (S141, K142, V145, N146, G147, G149, E150, L153, E154, M157, E158, Q161, L166) and highlighted red in Figure 4B. The relatively large CSD values for residues in EF4 are explained in part by the absence of a bound Ca^2+^ at EF4 in EF4mut (in contrast to Ca^2+^ bound at EF4 in Ca^2+^-saturated wildtype), which causes a change in the local charge environment in EF4. Therefore, large CSD values for residues in EF4 indicate a local main chain conformational change in EF4 due to Ca^2+^ binding at this site. However, mutagenesis of EF4 residues (F140A, K142D, L153R, L166R) shows almost no effect on cyclase activation (Table 1), suggesting these residues do not interact directly with RetGC1. We conclude that single mutations of EF4 residues having a relatively large CSD (F140A, K142D, L153R, L166R) are not sufficient to prevent Ca^2+^-induced conformational changes in EF4. 

**Table 1:** Mutagenesis of GCAP1 residues with high CSD values.

Amino Acid	CSD^1^	Mutation	RetGC1 A_max_^2^ (nmol cGMP/mg/min)	GC1 Affinity^2^, K_1/2_ (μM)
		Wild type	29.1 ±0.7 (N=2)	1.7 ±0.06
K23	0.06	D23	8.7 ±0.6 (N=2)	25 ±3
T27	0.16	E27	28.4 ±0.4 (N=2)	2.9 ±0.1
G32	0.14	N32	13.6 ±0.6 (N=2)	40 ±6.5
F43	0.06	A43	28.2 ±1.2 (N=2)	1.2 ±0.1
V77	NA^4^	E77	ND^3^ (N=3)	ND (N=3)
A78	NA	E78	ND (N=3)	ND (N=3)
L82	NA	E82	ND (N=2)	ND (N=2)
W94	NA	F94	15.1 ±0.7 (N=2)	7.3 ±1.1
I115	0.15	W115	25.6 ±0.6 (N=2)	2.4 ±0.1
F140	0.21	A140	29.5 ±0.4 (N=2)	1.7 ±0.02
K142	0.26	D142	27.8 ±0.6 (N=2)	1.3 ±0.03
S152	0.29	E152	20.3 ±0.5 (N=2)	7.3 ±0.9
L153	0.16	R153	27.4 ±0.1 (N=2)	3.2 ±0.07
L166	0.12	R166	27.6 ±0.5 (N=2)	3.9 ±0.2

1 Chemical shift difference (CSD) from values plotted in Figure 4A.

2 RetGC activity and apparent affinity for GCAP1 (mean±SD) determined as described in Methods.

3 ND: non-detectable.

4 NA: not assigned due to exchange broadening.

**Figure 5:**
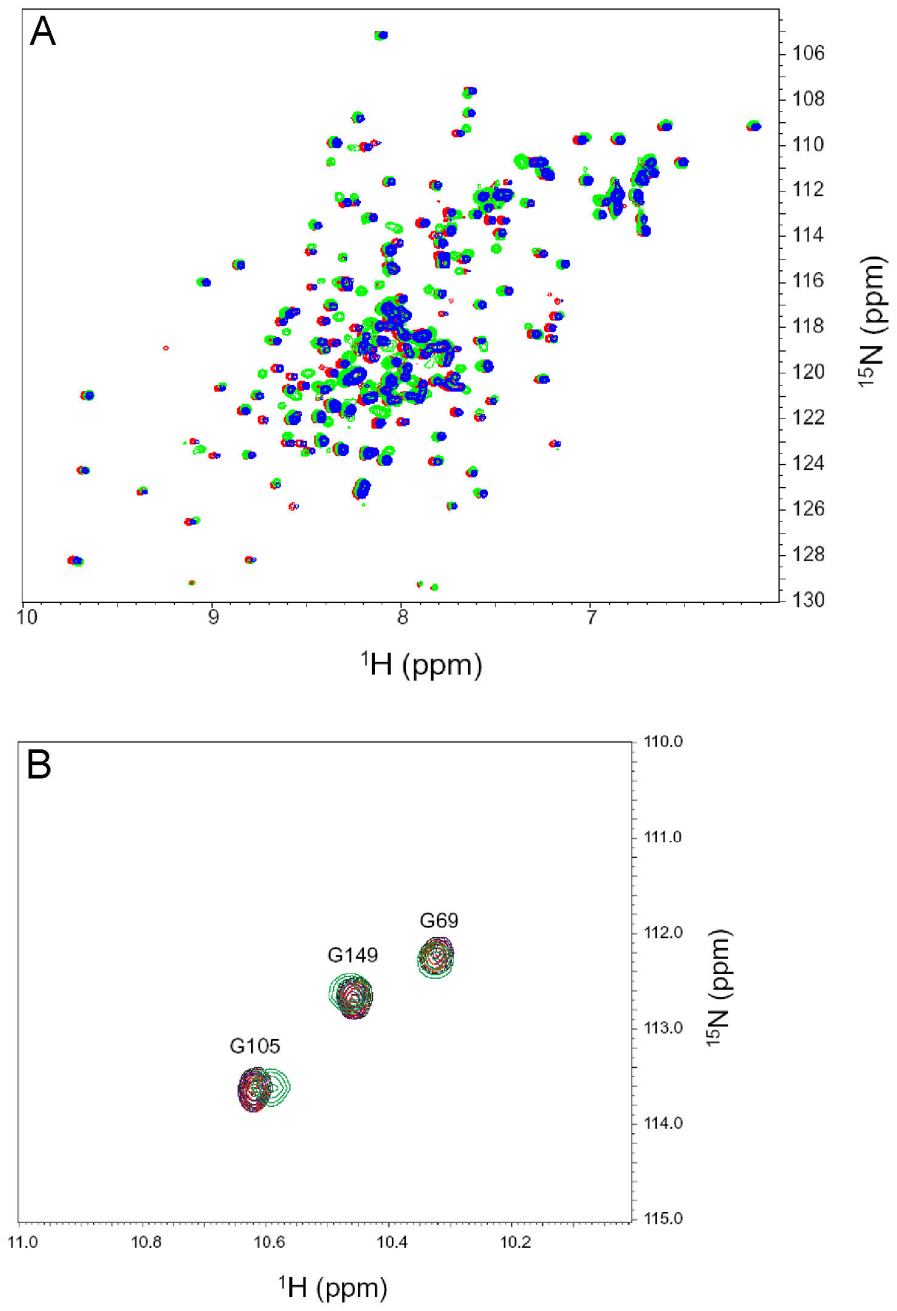
Structural characterization of GCAP1 mutants at the domain interface. (A) Overlay of ^15^N-^1^H HSQC spectra of V77E (red), L82E (blue), WT (black) and W94F (green) indicate that each mutant is properly folded and structurally intact. The mutant spectra look similar to that of wildtype particularly for residues in structured regions. Minor spectral differences are observed for exposed residues in unstructured regions, most likely due to small differences in solvent conditions. (B) Expanded view of ^15^N-^1^H HSQC downfield region for GCAP1 mutants: V77E (red), L82E (blue), WT (black) and W94F (green). Three downfield NMR peaks assigned to G69, G105 and G149 indicate Ca^2+^ is bound functionally at EF2, EF3 and EF4 for each mutant.

A few residues in EF1 (K23, T27, G32) have relatively high CSD values (Figure 4B), consistent with a structural change in EF1 even though EF1 does not bind Ca^2+^. Mutagenesis of some of these EF1 residues (K23D, G32N) caused a strong decrease in the ability of GCAP1 to activate cyclase with more than 10-fold decrease in binding affinity to the cyclase (Table 1 and Figure 5). However, there was no obvious correlation between the magnitude of the CSD and their importance for RetGC activation, because substitutions of T27 did not strongly affect activation of RetGC1. Nonetheless, the detected change in structural environment for some EF1 residues (K23, G32) would be consistent with EF1 undergoing a functional conformational change related to its activator state. The drastic decrease in apparent affinity of GCAP1 for RetGC1 caused by substitutions of K23 and G32 compared to that of wildtype GCAP1 argues that these EF1 residues are located at or near the RetGC binding site of GCAP, consistent with observations from previous mutagenesis studies [34,35]. Our use of GCAP1 and RetGC1 from different mammalian species should not affect the interpretation of the functional data, because mammalian GCAP1 homologs are nearly identical, use the same regulatory mechanisms, and work across different mammalian species in transgenic models [11,36–38].

### Residues at the GCAP1 domain interface have broad NMR resonances

The crystal structure of GCAP1 [21] contains two globular domains (N-domain: EF1 and EF2 and C-domain: EF3 and EF4) that form contacts involving residues in EF2 and EF3, called the domain interface. Residues at the GCAP1 domain interface (V77, A78, L82, W94, L98) and domain linker (residues S81 - Q90) have not been assigned in Figure 4A because their backbone resonances are broadened beyond detection. The broadening of these resonances is likely caused by exchange dynamics on the chemical shift time scale. Indeed, the corresponding residues in recoverin have been shown to undergo millisecond exchange dynamics [39] and recoverin’s domain interface region (exiting helix of EF2 and entering helix of EF3) is conformationally dynamic [40]. The millisecond dynamics of the domain interface in recoverin is a consequence of the Ca^2+^-induced domain swiveling that alters the interface between EF2 and EF3 [23]. We suggest that a similar conformational change (that alters contacts between EF2 and EF3) may also take place in GCAP1, but with perhaps a smaller overall displacement of the two domains. 

**Figure 6:**
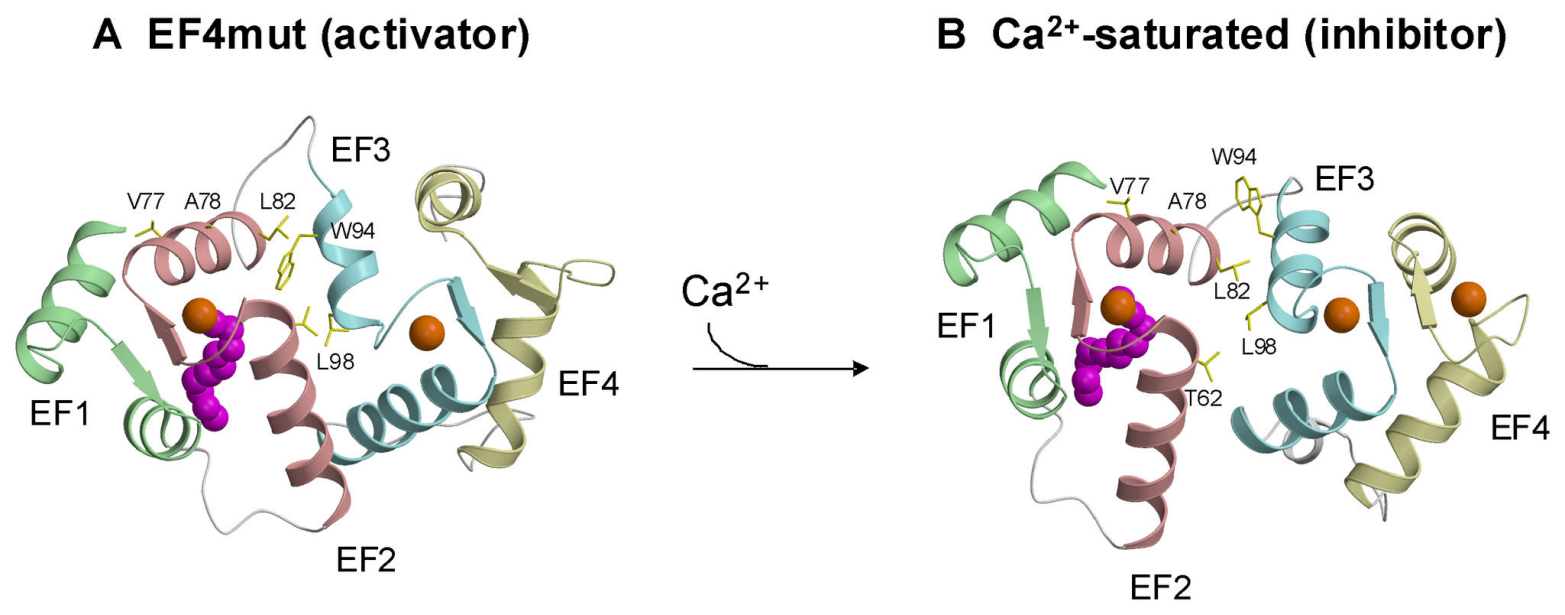
Schematic model of conformational changes in GCAP1 caused by Ca^2+^-binding at EF4. (A) Structural model of EF4mut activator state was generated by homology modeling using the NMR structure of recoverin (1jsa) that contains Ca^2+^ bound at EF2 and EF3 [23]. The four EF-hands are colored green (EF1), red (EF2), cyan (EF3) and yellow (EF4), and bound Ca^2+^ is orange. (B) The crystal structure of Ca^2+^-saturated GCAP1 (2r2i) shows key hydrophobic residues at the domain interface are solvent exposed in the Ca^2+^-bound inhibitor state. The Ca^2+^-dependent rearrangement of the W94 side-chain at the domain interface might control the switching between activator and inhibitor states. The N-terminal myristoyl group (magenta) is sequestered inside the protein in both structures.

Unlike recoverin that switches between stably folded Ca^2+^-free and Ca^2+^-bound conformational states, the tertiary structure of Ca^2+^-free/Mg^2+^-free GCAP1 forms a molten globule-like state [28], despite preserving most of its secondary structure [41]. Consequently, the apo form of GCAP1 is physiologically inactive [12,19,42]. Instead, GCAP1 always contains metal (Mg^2+^ or Ca^2+^) bound at EF2 and EF3 in the activator state, and Ca^2+^ binding at EF4 causes a functional transition to the inhibitor state [43]. To gain some insight into a possible conformational change in GCAP1 due to Ca^2+^ binding at EF4, we constructed a homology model for the EF4mut activator state (Ca^2+^ bound at EF2 and EF3) based on the known structure of recoverin that contains Ca^2+^ bound at EF2 and EF3 [23] (see Figure 6). The N-domain in the homology model (extruded myristoyl group) was replaced by the N-domain from the GCAP1 crystal structure (sequestered myristoyl group). In this structural model of EF4mut (with sequestered myristoyl group and Ca^2+^ bound at EF2 and EF3), the domain interface consists of residues in the exiting helix of EF2 (V77, A78 and L82) that are mostly solvent exposed and make contacts with residues in the entering helix of EF3 (W94 and L98). The modeled structure of EF4mut at the domain interface (Figure 6A) is similar to what is seen in the GCAP1 crystal structure (Ca^2+^-saturated inhibitor state, Figure 6B). However, a unique structural feature of EF4mut can be seen in the entering helix of EF3 that is shortened by a half turn, which alters the orientation of the W94 side-chain. In EF4mut (Figure 6A), the W94 indole ring points downward below L82, whereas the indole group points upward above L82 in the GCAP1 crystal structure (Figure 6B). The rearrangement of the W94 indole side-chain at the domain interface in activator vs inhibitor states of GCAP1 would kinetically alter the chemical shift environment in this region and might help explain the observed broadening of NMR resonances for V77, A78, L82 and W94.

If a conformational change at the domain interface is functionally relevant and directly controls cyclase activation, then one would expect specific mutations at the interface to have functional effects. We constructed single mutants of residues at the domain interface (V77E, L82E and W94F) that exhibit exchange broadened NMR resonances. Quite strikingly, we find that each one of these mutants of GCAP1 has lost the ability to activate RetGC (Table 1). Each mutant was verified by NMR to remain structurally intact as evidenced by the spectral similarity for residues in structured regions (Figure 5A), but interestingly, the V77E mutant (unlike wildtype) is monomeric even at high protein concentrations used for NMR (Figure 3C). The three downfield NMR resonances (at ~10.5 ppm in Figure 5B) for each mutant (V77E, L82E and W94A) show that Ca^2+^ is bound at EF2, EF3 and EF4, and that each mutant retains functional Ca^2+^ binding. Therefore, the lack of cyclase activation by these mutants is not due to protein misfolding. Our NMR and mutagenesis results demonstrate that residues at the domain interface in GCAP1 are conformationally dynamic, essential for activation of RetGC, and at least one (V77) is required for GCAP1 dimerization.

## Discussion

NCS proteins like recoverin and NCS-1 undergo large Ca^2+^-induced conformational changes that cause extrusion of the N-terminal myristoyl group, termed Ca^2+^-myristoyl switch [23,29,40]. A major difference in the structural dynamics between the different NCS proteins is the lack of a Ca^2+^-myristoyl switch in GCAP proteins [28,44,45], and different structural roles for myristoylation and metal binding to control membrane targeting [46,47]. Although crystal structures are known for Ca^2+^-bound GCAPs [21], little is known structurally about the Mg^2+^-bound/Ca^2+^-free activator state of GCAPs and how Ca^2+^-induced conformational changes in GCAPs control cyclase activation.

In this study, we present NMR assignments and mutagenesis data for GCAP1 to probe Ca^2+^-dependent conformational changes between the Ca^2+^-saturated inhibitory state versus the EF4mut activator state that contains Ca^2+^ bound at EF2 and EF3 (Figure 2) which mimics the activator state [12]. Overall, the NMR chemical shift differences between Ca^2+^-saturated wild type GCAP1 and EF4mut are relatively small (Figure 4), suggesting a similar overall main chain conformation for the two conformational states of GCAP1. This is in a sharp contrast to large Ca^2+^-induced conformational changes seen for the corresponding residues in other NCS proteins, including recoverin [23] and NCS-1 [29]. NMR relaxation data and size-exclusion chromatography analysis indicate that Ca^2+^-saturated GCAP1 and EF4mut (with Ca^2+^-bound at EF2 and EF3) are both dimeric in solution under NMR conditions (Figure 3). A GCAP1 homolog, GCAP2 undergoes a Ca^2+^-sensitive dimerization at micromolar protein concentrations, which originally suggested that reversible dimerization may control formation and activation of RetGC:GCAP in a 2:2 complex [35,48]. Although dimerization of GCAP1 observed in this study (at high protein concentration) does not appear to be Ca^2+^ sensitive, it is possible that GCAP1 dimerization might promote a functional interaction within a RetGC1 dimer on the disk membrane [49,50]. The binding stoichiometry of the GCAP1:RetGC1 complex has been estimated to be equimolar [51], consistent with a complex that contains a RetGC1 dimer bound to two molecules of GCAP1. We show in this study that a GCAP1 mutation (V77E) that eliminates GCAP1 dimerization (Figure 3C) also abolishes its ability to activate RetGC (Table 1). However, it is not clear whether the V77E mutation in GCAP1 disrupts its primary binding to RetGC or prevents secondary interactions within the RetGC1/GCAP1 complex.

Our NMR and mutagenesis data indicate that some residues in EF1 of GCAP1 undergo a conformational change that controls activation of RetGC. Although GCAP1 does not exhibit a large main-chain conformational change like in recoverin, GCAP1 residues in EF1 (K23, T27, G32) and EF4 show relatively large backbone chemical shift differences between Ca^2+^-saturated GCAP1 and EF4mut (Figure 4). Mutagenesis of some EF1 residues (K23D and G32N) shows relatively large effects on RetGC activation and binding (Table 1). The decreased affinity for cyclase binding by both mutants should prevent their binding to the cyclase under in vivo conditions, because the estimated concentration of GCAPs and RetGC isozymes in the rod outer segment does not exceed 10 μM [52,53]. 

Previous studies have implicated EF1 as a possible binding site for the cyclase [14,35,54,55]. Indeed, our observation in this study that Ca^2+^-binding at EF4 can remotely affect the structure of EF1 is consistent with the recently proposed Ca^2+^-myristoyl tug mechanism [56]. The binding of either Mg^2+^ or Ca^2+^ to EF2 and EF3 is sufficient for GCAP1 binding to RetGC1 [42] and promoting the proper conformation of GCAP1 in the activator state [12]. Therefore, Ca^2+^ binding to EF4 is the main determinant that switches GCAP1 from activator to inhibitor state. It is possible that Ca^2+^-binding to EF4 causes local structural changes in EF4 (see large CSD values for EF4 residues in Figure 4) that can be transmitted to EF1 through the N-terminal myristoyl group and C-terminal helix in GCAP1 (residues M165 – Q187). Indeed, assigned residues in the C-terminal helix (L176, T177, I179) exhibit large CSD values (Figure 4A) and are conformationally dynamic (Figure 3B). Thus, the structural link between the myristoyl group and both EF1 and the C-terminal helix may serve as a lever that connects the observed conformational changes in EF1 and EF4 (Figure 4). 

 Several studies suggest a possible role for EF1 in forming the RetGC1 binding interface. Point mutations of several EF1 residues in GCAP2 can suppress its ability to bind and activate RetGC1 [35], and deletion of the conserved Cys and Pro in the EF1 loop also suppresses GCAP1’s ability to stimulate RetGC1 [34]. The replacement of the EF1 domain in GCAP2 or GCAP1 with that of recoverin or neurocalcin eliminates their ability to activate the cyclase [14,54]. However, the structural determinants and dynamics in EF1 that control the activator-to-inhibitor transition of GCAP1 are still poorly understood and will require further investigation.

GCAP residues in EF2 and EF3 located at the domain interface (V77 – W94, see Figures 1 and 6) appear to be another “hot spot” for controling activation of RetGC [14,54]. GCAP1 residues at the domain interface have quite broad NMR resonances that are temperature sensitive. The corresponding residues in recoverin (Figure 1) also exhibited broad NMR resonances and ^15^N NMR relaxation dispersion studies reveal that these domain interface residues are conformationally dynamic [39]. Ca^2+^-induced rearrangement of residues at the domain interface in recoverin gives rise to a 45 degree swiveling of the two domains [23]. Our homology model of EF4mut suggests a related but much smaller Ca^2+^-induced structural change at the domain interface in GCAP1 (Figure 6). The most noteworthy Ca^2+^-induced structural difference in GCAP1 can be seen in the entering helix of EF3 that unravels a half turn in EF4mut, which causes a repositioning of the W94 side-chain at the domain interface. A Ca^2+^-induced change in the structural environment around W94 is consistent with previous tryptophan fluorescence and electron paramagnetic resonance studies of GCAP1 [12,19,57]; however, mutations of W94 do not significantly alter the Ca^2+^-binding affinity of GCAP1 [12]. We suggest that exposure of W94 indole side-chain in the GCAP1 activator state might play a role in contacting RetGC1. 

Other hydrophobic residues at the GCAP1 domain interface (V77, A78, L82) remain solvent exposed in both the EF4mut (Figure 6A) and Ca^2+^-saturated crystal structure (Figure 6B). The exposure of these hydrophobic residues might mediate specific contacts with RetGC and/or control GCAP1 dimerization. Indeed, mutagenesis of GCAP1 residues at the domain interface both disrupts GCAP1 dimerization (Figure 3C) and abolishes activation of the cyclase (Table 1). Future studies are needed to better understand how residues at the domain interface control GCAP1 dimerization and/or modulate interactions with RetGC.

The structure and dynamics of the important C-terminal region of GCAP1 (residues 170 - 175) could not be examined in our study, because most of the NMR resonances in this region were exchange broadened. In GCAP2, Ca^2+^ binding makes its C-terminal phosphorylation site more accessible for cGMP-dependent kinase [58]. However, we presently cannot draw any conclusion about the structural dynamics of residues, L170-D175 in GCAP1, because there is no such phosphorylation site in GCAP1. Also, we could not determine CSD values for these residues due to the broadening of NMR resonances in this region for both the activator and inhibitor states.

 One of the most important outstanding questions for understanding GCAP structure-function relationships is to determine the location of the cyclase binding site in the GCAPs. Previous studies [14,35,54] and the current study indicate three possible regions in GCAPs that control their ability to activate RetGC: EF1, the interface between EF2 and EF3, and the C-terminal region adjacent to EF4. The CSD detected by NMR spectroscopy reveal conformational changes related to some of these residues in GCAP1. Importantly, most of the conformational changes observed in this study for EF4mut may be similar to those caused by a mutation, E155G, triggering congenital retinal dystrophy in human patients. This mutation also prevents Ca^2+^ binding to EF4 and, by constitutively activating RetGC1, produces excessive accumulation of cGMP and Ca^2+^ in photoreceptors thus causing their apoptotic death [20,38]. Moreover, many other mutations cause congential human blindness by affecting Ca^2+^ binding to EF4 [41,59–61]. Therefore, the use of CSD in EF4mut as a model provides a useful tool for evaluating the structural changes in GCAP1 upon its conversion into the constitutively active state. 

However, CSD alone is not sufficient to identify specific residues that make direct contact with the cyclase, because the CSD does not discriminate between backbone changes caused by the Ca^2+^ sensor action versus backbone changes required to accommodate RetGC1 binding. Some of the residues (e.g. K23 and G32 in EF1) display CSD that may reflect their potential involvement in the cyclase binding interface. However, for most residues that exhibited large CSD, mutations altering the properties of the side chain did not abolish cyclase activation (Table 1). Hence, the most prominent CSD observed for residues in EF3 and EF4 (which are still rather small compared to the CSD observed in recoverin) may simply reflect localized metal-dependent conformational changes that occur outside the cyclase-binding site. Thus, the mapping of the cyclase-binding site in GCAP1 cannot be derived from the CSD alone. A comprehensive mutagenesis of exposed surface residues in GCAP1 is currently under way to pinpoint those residues mapped onto the GCAP1 structure (Figure 6) that are directly involved in forming the cyclase-binding interface.

## Materials and Methods

### Expression and purification of GCAP1 and mutants

Mutations were introduced in a bovine GCAP1 coding plasmid using a “splice by overlap extension” approach as previously described [11]. Myristoylated GCAP1 and its mutants were produced in *E. coli* strain harboring yeast N-myristoyl transferase and purified to homogeneity using previously described method [12]. The expression and purification of isotopically labeled GCAP1 and mutants were described previously [28]. Uniformly ^15^N-labeled GCAP1, ^15^N-labeled EF4mut, and triple labeled ^2^H/^15^N/^13^C-labled EF4-mut used in the NMR studies (0.5 mM) were dissolved in 5 mM Tris-*d*_11_ (pH 7.4), 5 mM CaCl_2_, 5 mM MgCl_2_, 5 mM dithiothreitol-_d10_, 40 mM octyl-β-D-gluco-pyranoside and 93%/7% H_2_O/D_2_O.

### Guanylyl cyclase assay

Recombinant human RetGC1 was expressed in HEK293 cells and assayed *in vitro* using [α-^32^P] GTP as a substrate as previously described in detail [12,56]. Ca/Mg/ethylene glycol tetraacetic acid buffers for the assay were prepared and calibrated as previously described [12].

### NMR spectroscopy

NMR experiments and backbone assignments for Ca^2+^-saturated GCAP1 were described elsewhere [31]. Backbone NMR resonance assignments of the EF4mut activator state (Ca^2+^ bound at EF2 and EF3) were obtained in this study by the analysis of three-dimensional NMR data as described previously [62]. 2D ^15^N-^1^H HSQC with 2048 (^1^H) x 256 (^15^N) data points, 3D HNCACB with1024 (^1^H) x 64 (^15^N) x 120 (^13^C) data points, and 3D HNCO with 2048 (^1^H) x 64 (^15^N) x 128 (^13^C) data points were all performed on a triple-labeled EF4mut sample using 800 MHz Bruker NMR spectrometer equipped with a triple resonance cryogenic probe. In addition, 3D HNCOCACB with 1024 (^1^H) x 64 (^15^N) x 128 (^13^C) data points was performed on the triple-lableled sample using a 600 MHz Bruker NMR spectrometer, equipped with a triple resonance cryogenic probe. All NMR experiments were performed at 37 °C. Spectra were processed using NMRPipe software package [63] and analyzed using SPARKY.

### ^15^N NMR relaxation analysis

^15^N NMR longitudinal relaxation rates (R_1_) and transverse relaxation rates (R_2_) of Ca^2+^-saturated GCAP1 and EF4mut were measured using pulse schemes described previously at 60.81 MHz ^15^N resonance frequency [64]. Relaxation delays for R_1_ experiments were 0.01, 0.25, 0.5, 1.0 and 2.0 s. Relaxation delays for R_2_ experiments were 8, 16, 24, 32, 48, 64 and 80 ms. The 180° pulses for Car-Purcell-Meiboom-Gill (CPMG) sequence were applied every 1.0 ms. Uncertainty of R_1_ and R_2_ values were determined by Monte-Carlo error simulation using signal intensities that contain experimental noise fluctuation. Model-free parameters including generalized order parameters and correlation times for internal motion [65–67] were determined using the protocol described previously [64]. 

### Size exclusion chromatography and multi-angle light scattering

Determination of the molar mass of GCAP1 was carried out using a multi-angle light scattering instrument (Wyatt Technologies Inc. TREOS and Optlab Rex) coupled to a Superdex 200 HR 10/30 column (Amersham) at 4 °C equilibrated in buffers containing either 10 mM phosphate (pH 7.0) or 10 mM acetate (pH 4.5). A 0.1 ml aliquot of protein (200 μM) was loaded onto the column and eluted at a flow rate of 0.5 ml/min. The molar mass of the eluted protein was calculated from the observed light scattering intensity as described previously [68]. Apparent molecular weights were also calculated using a standard curve of V_e_/V_o_ versus the log of the molecular weights of standard proteins: β-amylase (200 kDa), alcohol dehydrogenase (150 kDa), carbonic anhydrase (29 kDa), and cytochrome c (12.4 kDa). V_o_ is a void volume obtained using blue dextrane (2000 kDa) and V_e_ is a volume of elution. 

### Homology model structure calculation

To generate a homology modeled structure of GCAP1 with Ca^2+^ bound at EF2 and EF3, we first used the NMR structure of Ca^2+^-bound recoverin (1JSA) that contains Ca^2+^ bound at EF2 and EF3 as a template in the modeler software, Swiss-Model [69]. The resulting homology modeled structure contained an extruded myristoyl group, which disagrees with the myristoyl group being sequestered inside GCAP1 in both the activator and inhibitor states [28]. Therefore, we replaced the N-terminal domain of the homology modeled structure (residues 2-87) with the structure of the corresponding residues from the GCAP1 crystal structure (2R2I) that contains a sequestered myristoyl group. The main chain atoms of the N-terminal domain from the GCAP1 crystal structure (residues 2-87) were superimposed onto the corresponding atoms in the homology modeled structure using the software, Chimera. This alignment procedure positioned the N-terminal domain of the GCAP1 crystal structure in close proximity to the C-terminal domain of the homology modeled structure (Ca^2+^ bound at EF3 and not bound at EF4). The last residue of the overlaid N-terminal domain (K87) was covalently attached to residue V88 from the homology modeled structure (using the patch command in Chimera), which effectively connected the N-terminal domain of 2R2I with the C-terminal domain generated by homology modeling. The resulting structure contained a sequestered myristoyl group with Ca^2+^ bound at EF2 and EF3. The entire modeled structure was energy minimized using Xplor-NIH. 
